# Fingerprinting 2,3,7,8-tetrachlorodibenzodioxin contamination within the lower Passaic River

**DOI:** 10.1002/etc.2961

**Published:** 2015-05-05

**Authors:** James D Quadrini, Wen Ku, John P Connolly, Deborah A Chiavelli, Peter H Israelsson

**Affiliations:** † Anchor QEAWoodcliff Lake, New Jersey, USA; ‡ Anchor QEABoston, Massachusetts, USA

**Keywords:** Dioxin, Superfund site, Fingerprint, 2,3,7,8-Tetrachlorodibenzodioxin, Passaic

## Abstract

The lower Passaic River is an operable unit of the Diamond Alkali Superfund site at 80 and 120 Lister Avenue in Newark, New Jersey, USA. Between 1948 and 1969, the Diamond Shamrock Chemicals Company and its predecessors manufactured chemicals such as pesticides and phenoxy herbicides, including 2,4,5-trichlorophenol, which is a precursor to 2,4,5-trichlorophenoxyacetic acid, one of the primary components used to make the military defoliant Agent Orange. A by-product of this manufacturing process was 2,3,7,8-tetrachlorodibenzodioxin (2,3,7,8-TCDD), and the site is considered the dominant source of 2,3,7,8-TCDD to the lower Passaic River and its environs. Several investigators have identified the ratio of 2,3,7,8-TCDD to total TCDD as a fingerprint for the site source. The present study presents data that establish polychlorinated dibenzodioxin/polychlorinated dibenzofuran (collectively, PCDD/F) congener and homolog fingerprints of soil and sump samples from the site. It then compares those fingerprints to the PCDD/F congener and homolog patterns in lower Passaic River sediments. The similarity of the patterns in lower Passaic River sediments to the site fingerprint indicates the site is the dominant source of the 2,3,7,8-TCDD in sediments within approximately the lower 14 miles of the lower Passaic River, excluding, for the purposes of the present discussion, Newark Bay. In addition, PCDD/F congener data indicate that the ratio of 1,3,7,8-TCDD to 2,3,7,8-TCDD is another marker of the site and corroborates the findings from the other fingerprints. *Environ Toxicol Chem* 2015;34:1485–1498. © 2015 The Authors. Published by SETAC.

## INTRODUCTION

The lower Passaic River is a 17.4-mile long, partially mixed estuary located in northern New Jersey, USA, extending from the Dundee Dam to Newark Bay (Supplemental Data, Figure S1). Beginning with cotton mills, industry in the lower Passaic River watershed grew to include manufactured gas plants; petroleum refineries; tanneries; shipbuilding, smelting, pharmaceutical, electronic product, dye, paint, pigment, paper, and chemical manufacturing plants; and other industrial activity 1,2. Major population centers such as Paterson and Newark transformed the watershed into a mix of residential, commercial, and industrial uses. Thus, like many other urban systems, the lower Passaic River has been subjected to contaminant loadings from a variety of public and private sources. A distinguishing characteristic of the lower Passaic River is elevated levels of 2,3,7,8-tetrachlorodibenzodioxin (2,3,7,8-TCDD) in sediments, to a degree that is atypical of most other urban water bodies.

The lower Passaic River is an operable unit of the Diamond Alkali site at 80 and 120 Lister Avenue in Newark, New Jersey, USA. The Diamond Alkali site, also known as the Lister Avenue site, is situated along the southern shore of the lower Passaic River, approximately 3.1 miles upstream (alternately landward or up-estuary) of where the lower Passaic River meets Newark Bay (Supplemental Data, Figure S1). Between 1948 and 1969, the Diamond Shamrock Chemicals Company and its predecessors manufactured chemicals such as pesticides and phenoxy herbicides, including 2,4,5-trichlorophenol, which is a precursor to 2,4,5-trichlorophenoxyacetic acid, one of the primary components used to make the military defoliant Agent Orange 3–7. During this time, process wastes from the site operations were discharged to the Passaic River 8,9. The site was placed on the Superfund National Priorities List in September 1984 because of 2,3,7,8-TCDD contamination, a by-product of the 2,4,5-trichlorophenol manufacturing process, found in on-site and off-site soils and groundwater 7. Several investigators have concluded that the site is the dominant 2,3,7,8-TCDD source to the lower Passaic River and its environs 3,5,10,11, as well as a significant historical source of dichlorodiphenyltrichloroethane 3,12. Bopp et al. 3 based this conclusion on spatial and temporal trends in 2,3,7,8-TCDD concentrations in regional sediments. Chaky 5 observed similar patterns in 2,3,7,8-TCDD concentrations as well as for the ratio of 2,3,7,8-TCDD to total TCDD, which is known to be characteristically high for waste associated with the 2,4,5-trichlorophenoxyacetic acid manufacturing processes used at the site. Soil samples collected in the vicinity of the site exhibited 2,3,7,8-TCDD to total TCDD ratios in the range of 0.86 to 0.98 13,14, and lower Passaic River sediments typically exhibit ratios in excess of 0.6 (discussed in the present study). In contrast, wastewater and atmospheric sources exhibit a much lower ratio, on the order of 0.06 or less 5.

The dominance of the site source has been questioned by several reports that rely on data from the 1990s and statistical pattern recognition techniques (e.g., principal components analysis, polytopic vector analysis, and cluster analysis) to suggest that polychlorinated dibenzodioxin/polychlorinated dibenzofuran (collectively, PCDD/F) contamination in the lower Passaic River and surrounding region comes from a multitude of sources [14–19]. Wenning et al. 14 further indicate that PCDD/F distributions in site soils and lower Passaic River sediments adjacent to the site are dissimilar to sediments elsewhere in the lower Passaic River. The conclusions of all of these reports are heavily influenced by dominant regionally contributed congeners (1,2,3,4,6,7,8-heptachlorodibenzodioxin [1,2,3,4,6,7,8-HpCDD] and octachlorodibenzodioxin [OCDD]) and take no account of concentration patterns (i.e., all fingerprints were normalized to remove any influence of concentration). They also take no account of a distinguishing characteristic of the site source, which is the dominance of 2,3,7,8-TCDD relative to total TCDD concentration and the characteristic fingerprint used by Chaky 5.

Two of the above studies 10,19 have also suggested a second 2,3,7,8-TCDD source located approximately 11 miles upstream of the lower Passaic River mouth on the Third River (Hansen 10 identified this source as the Givaudan Chemical Company facility, hereafter referred to as the Givaudan facility). However, this presumption is based on a single data point having congener concentrations an order of magnitude lower than the downstream sediments and provides no indication of a substantive impact on 2,3,7,8-TCDD concentrations in the river. A third study, by Garvey et al. 20, examined 2,3,7,8-TCDD concentration profiles, 2,3,7,8-TCDD to total TCDD ratios, and 2,3,7,8-TCDD to 4,4′-dichlorodiphenyldichloroethylene ratios in 5 dated sediment cores from the lower Passaic River and concluded that a second source of 2,3,7,8-TCDD exists in the vicinity of the Third River. Garvey et al. 20 acknowledged, however, that the magnitude of this source and its area of influence are almost certainly less than those from the downstream Lister Avenue site source.

The upland portion of the site underwent several remedial actions under New Jersey Department of Environmental Protection and US Environmental Protection Agency (USEPA) oversight between 1984 and 2004 21,22. A remedial investigation and feasibility study was initiated for the lower 6 miles of the lower Passaic River in 1994 and later expanded in 2003 to include the full 17.4 miles of the lower Passaic River. In 2004, a remedial investigation and feasibility study for Newark Bay, including portions of the Hackensack River, Arthur Kill, and Kill Van Kull, was initiated. Sediment chemistry data from the Passaic River and Newark Bay studies indicate that 2,3,7,8-TCDD is present within the lower Passaic River at levels that are much higher than those found in other regional sediments (i.e., those in the Passaic River above the Dundee Dam, in lower Passaic River tributaries, and in Newark Bay 23). In 2012, Occidental Chemical and Tierra Solutions removed approximately 40 000 cubic yards of lower Passaic River sediments containing 2,3,7,8-TCDD levels as high as 35 mg/kg from the immediate vicinity of the site (i.e., the phase 1 removal area shown in Supplemental Data, Figure S1). Also, Occidental Chemical and Tierra Solutions agreed to remove an additional 160 000 cubic yards of lower Passaic River sediments from an adjacent shoreline area on either side of the phase 1 removal area (i.e., the phase 2 removal area) 21. At the time of the present writing, Occidental Chemical and Tierra Solutions have not started removing sediments from the phase 2 removal area.

Fingerprinting is commonly used to distinguish contributions of contaminants among multiple sources and involves the examination of environmental data for a unique chemical concentration pattern that is attributable to a specific source. For PCDD/Fs, a fingerprint refers to the relative abundance of specific congeners or homolog groups, which, if sufficiently distinctive, can be used to identify sources even when the spatial concentration patterns for individual congeners, homolog groups, or total PCDD/Fs are inconclusive. Several investigators have identified the ratio of 2,3,7,8-TCDD to total TCDD as a fingerprint for the site source 5,11,23,24. The present study uses more comprehensive recent and historical PCDD/F measurements from lower Passaic River sediments and the site (see Table1 for number of sediment cores used in the present evaluation) to extend the analyses done by others, which relied on limited data sets, to examine the importance of the site to the 2,3,7,8-TCDD contamination within the lower Passaic River. It includes the results of a focused high-resolution sediment-coring program that was conducted to better understand the timing and magnitude of 2,3,7,8-TCDD discharges from the site. Together, these data provide a basis to examine the temporal and vertical distribution of 2,3,7,8-TCDD concentrations and PCDD/F composition and are used to demonstrate the similarity of PCDD/F patterns in near-site sediments to samples collected on-site. A representative PCDD/F congener fingerprint and a representative PCDD/F total homolog fingerprint of the site were identified along with 2 characteristic markers, the ratio of 2,3,7,8-TCDD to total TCDD and the ratio of 1,3,7,8-TCDD to 2,3,7,8-TCDD. The consistency of these data with PCDD/F patterns in sediments collected throughout the lower Passaic River is considered, and the utility of the 2 congener ratios as tracers of the site discharge is demonstrated. Potential impacts of the site discharges on areas outside of the lower Passaic River (e.g., Newark Bay) are not addressed in the present study.

**Table 1:** Summary of core counts used in the peak 2,3,7,8-tetrachlorodibenzodioxin (2,3,7,8-TCDD) fingerprinting analyses

Study	Total no. of cores	No. of cores with definitive 2,3,7,8-TCDD concentration peaks
2008 Low-resolution coring program	107	100
2009 Tierra Solutions sediment cores	12	12
2011 High-resolution cores sampling	3	3
2011/2012 River mile 10.9 characterization program	69	69
2012 Supplemental sampling program	85	46
2013 Second supplemental sampling program	74	40

## MATERIALS AND METHODS

### Data sets

Several data sets were considered. A brief description of each is provided below.

#### 1990/1992 Lister Avenue site data

Three surface soil samples and 1 sump sample were collected from the site in October 1990. Samples A1 and A3 were collected adjacent to the former processing building where 2,4,5-trichlorophenol was manufactured, whereas sample A2 was collected closer to the former chemical manufacturing building (Supplemental Data, Figure S1). In January 1992, 4 samples were collected from the site: 2 sump residue samples, 1 tetrachlorobenzene tank residue sample, and 1 tetrachlorobenzene tank unloading line sample (samples A4–A7 in Supplemental Data, Figure S1). All samples were collected prior to site remediation and analyzed for the 17 2,3,7,8-substituted PCDD/F congeners. The tetrachlorobenzene tank residue sample (A6) and the tetrachlorobenzene tank unloading line sample (A7) were not included in the present analysis because these samples were collected at locations in the process prior to 2,4,5-trichlorophenol manufacturing. The soil and other samples collected from the site are likely more representative of the different stages of the manufacturing process, different points in the disposal processes at the site, and the events and processes following the plant explosion and main processing facility replacement in the early 1960s.

#### 2009 Tierra Solutions sediment cores

Twelve sediment cores were collected from within the phase 1 removal area by Tierra Solutions as part of the phase 1 removal action predesign activities 25. The locations of these cores are shown in Supplemental Data, Figure S1. At each location, cores were collected to a depth of 12 feet below the sediment surface, sectioned in 2-foot intervals (i.e., 0–2 feet, 2–4 feet, etc.), analyzed for the 17 2,3,7,8-substituted PCDD/F congeners, and validated per the USEPA-approved Quality Assurance Project Plan 25. The PCDD/F homolog results were reported for only 3 of the homolog groups. These data were used to assess sediment chemistry within the phase 1 removal footprint prior to removal.

#### 2011 high-resolution core sampling

Three high-resolution cores (HRCs) were collected from within the phase 1 removal area (prior to removal) within the lower Passaic River immediately adjacent to the site in January 2011 by Woodard and Curran on behalf of the Joint Defense Group and Lower Passaic River Small Parties Group, Newark, NJ, USA. Two of the cores (HRC-02H and HRC-04H) were situated close to the shoreline, whereas the third (HRC-03H) was located farther away from the shoreline (Supplemental Data, Figure S1). The cores were sectioned in 2-inch intervals, a subset of which was submitted for high-resolution PCDD/F congener analysis (via method 1613B), cesium-137, lead-210, various other analytes, and physical properties 26,27. All results underwent full level IV data validation in October 2011 following USEPA Region 2 Data Validation standard operating procedures 28. Some PCDD/F congener results were estimated by the laboratory because of matrix interferences from very high concentrations of 2,3,7,8-TCDD, which caused percentage recoveries of the coeluting cleanup standard component measurements to fall outside of laboratory control limits; these results were deemed acceptable by the laboratory and suitable for the purpose of the analyses presented herein. The data for the present study were collected, processed, analyzed, and validated consistent with the USEPA-approved Quality Assurance Project Plan 27.

#### Lower Passaic River remedial investigation and feasibility study data sets

Numerous sediment-sampling activities have been performed as part of the ongoing lower Passaic River remedial investigation and feasibility study by the Lower Passaic River Cooperating Parties Group (Newark, NJ, USA) to characterize the physical and chemical properties of the lower Passaic River sediments. The present study considers sediment samples collected as part of the following programs: 2008 low resolution coring sediment sampling program (107 cores) 27; 2009/2010 remedial investigation field sampling plan 2 benthic sediment sampling program (132 surface [0–6 inches] grabs) 29; 2012 supplemental sediment sampling program (85 cores) 30; and 2013 second supplemental sampling program (66 cores and 8 surface [0–6 inches] grabs) 31.

The cores collected as part of the 2012 supplemental sediment sampling program were considered short cores because they generally targeted select sediment intervals at each location (i.e., mostly 0–6 inches, 6–18 inches, and 18–30 inches), not the full sediment column 30. The 2013 second supplemental sediment sampling program cores were collected and sectioned in a similar manner as the 2012 supplemental sediment sampling program cores, except the 2013 second supplemental sediment sampling program cores also collected the final 1-foot interval above native material or refusal 31. These remedial investigation and feasibility study data sets were selected because of their comprehensive spatial coverage throughout the lower Passaic River. The data for these programs were collected, processed, analyzed, and validated under USEPA-approved Quality Assurance Project Plans 27,29,30.

In addition to the above remedial investigation and feasibility study data sets, sampling results from a focused investigation of an approximate 8.9-acre fine sediment area located along the eastern shoreline near Lyndhurst, New Jersey (Supplemental Data, Figure S1)—2011/2012 River Mile (RM) 10.9 Characterization Program—were included in the present evaluation. This area was subject to a detailed preremediation sediment characterization because of elevated levels of 2,3,7,8-TCDD and other contaminants found there 32. In 2011, sediment cores were collected at 54 locations to characterize the nature and extent of contaminated sediments 32. In 2012, an additional 15 locations were sampled to characterize sediments along the shore 33. The sediment cores were sectioned 0 inches to 6 inches for the surface layer, 1-foot intervals for intermediate depths (i.e., 0.5–3.5 feet), and 2-foot intervals for depths greater than 3.5 feet. Samples were analyzed using high-resolution PCDD/F congener analysis 32. In fall 2013, the Lower Passaic River Cooperating Parties Group commenced a sediment removal action from a 5.6-acre portion of the deposit 33. This work was completed in spring 2014.

#### Nonremedial investigation and feasibility study data sets

The lower Passaic River has been subject to extensive study since the 1980s. Numerous sediment sampling activities have been conducted in the lower Passaic River but are not included in the present evaluation because of objectives that differ from those data sets collected under the lower Passaic River remedial investigation and feasibility study, incomplete spatial coverage, or data quality concerns associated with older analytical techniques and methods.

### Summary of sediment data treatment

The present study presents spatial trends in the ratio of various PCDD/F congeners, average PCDD/F congener, and homolog fingerprints from throughout the lower Passaic River and vertical concentration and PCDD congener ratios from the 2011 HRCs. For all data sets, PCDD/F concentrations are presented as reported by the laboratories (i.e., unadjusted). This includes the 2008 low-resolution coring sediment sampling program conducted as part of the lower Passaic River remedial investigation and feasibility study, where the USEPA, through an analysis of sediment split samples, determined that correction factors should be applied to this PCDD/F congener data set because of a low bias in the reported values 34. These correction factors were only prescribed for the 17 2,3,7,8-substituted PCDD/F congeners; correction factors for PCDD/F homologs were not provided. A parallel set of analyses (i.e., similar to those in the present study) was conducted using the bias-corrected PCDD/F concentrations, and the parallel analyses showed that the results of the evaluation presented are not significantly affected by the bias correction. Thus, for consistency across all data sets presented, only raw unadjusted concentrations were used.

Spatial trends are analyzed for surface sediment and peak sediment PCDD/F concentrations. Surface sediment PCDD/F concentrations and congener ratios are from samples collected from within the top 6 inches of the sediment column, whereas peak sediment PCDD/F concentrations and congener ratios are the results observed at the local maximum 2,3,7,8-TCDD concentration for each of the 2008 low-resolution coring sediment sampling program, 2009 Tierra Solutions, 2011 HRC, 2011/2012 RM 10.9, 2012 supplemental sediment sampling program, and 2013 second supplemental sediment sampling program locations. The 2009/2010 remedial investigation field sampling plan 2 benthic sediment sampling program was not included in the peak data set because it was limited to the top 6 inches of the sediment column. The 2,3,7,8-TCDD concentration profiles for each core were examined to identify those with a 2,3,7,8-TCDD concentration peak. Cores were deemed usable for this analysis if they were at least 2.5 feet deep; had at least 3 segments, including 1 at the surface; had gaps between sediment core intervals with measured concentrations that were less than 0.1 feet; and met at least 1 of the following criteria: the maximum 2,3,7,8-TCDD concentration is not in the bottom segment, the maximum 2,3,7,8-TCDD concentration is greater than 10 000 ng/kg, and the 2,3,7,8-TCDD concentration in the bottom segment is less than 30 ng/kg. These criteria were established to identify cores that likely captured most or all of the 2,3,7,8-TCDD inventory, and a representative distribution of 2,3,7,8-TCDD concentration from the sediment surface to native material or the depth of refusal was obtained. In a few instances, cores not meeting the criteria described above were included based on visual inspection of the 2,3,7,8-TCDD concentration profiles. In all other instances, cores not meeting these criteria were excluded from the analysis of peak concentration trends but were still considered for the surface sediment trend evaluations. The number of cores used in the peak concentration evaluations is summarized in Table1.

For congener ratio analyses, samples where either PCDD/F congener (i.e., the numerator or denominator) were reported below the detection limit were excluded to avoid uncertainty associated with the actual concentration of 1 or both of these congeners. For the PCDD/F congener and homolog fingerprinting comparisons, nondetect PCDD/F congener concentrations were set to 0 prior to averaging. The fingerprints presented in the present study were not sensitive to the assumption of how to quantify congeners reported as nondetects (results not shown). Samples with 2,3,7,8-TCDD concentrations reported below the detection limit or where more than half of the 15 PCDD/F congeners (excluding 1,2,3,4,6,7,8-HpCDD and OCDD, see discussion below) were reported below the detection limit were excluded from the fingerprint analyses. In addition, samples from various stretches of the lower Passaic River were grouped prior to averaging. This was done to examine large-scale longitudinal trends and to facilitate discussion. The stretches presented include RM 0 to RM 8, RM 8 to RM 14, RM 14 to RM 17.4, and RM 17.4 to RM 18. The breakpoints used in the analysis were selected as follows: RM 8 was based on the approximate upstream limit that the USEPA considered in its focused feasibility study 1 and corresponds approximately to where the lower Passaic River transitions to primarily silt (i.e., below RM 8); RM 14 was taken as the approximate upstream extent of the site discharge influence 23; RM 17.4 was based on the location of the Dundee Dam, which provides a physical barrier to upstream transport from the lower Passaic River to the upper Passaic River; and RM 18 was based on the upstream extent of sediment data collected as part of the lower Passaic River remedial investigation and feasibility study. Because of the density of samples collected from the RM 10.9 sediment deposit, these data were not included in the RM 8 to RM 14 bin; rather, they were evaluated separately.

**Figure 1:**
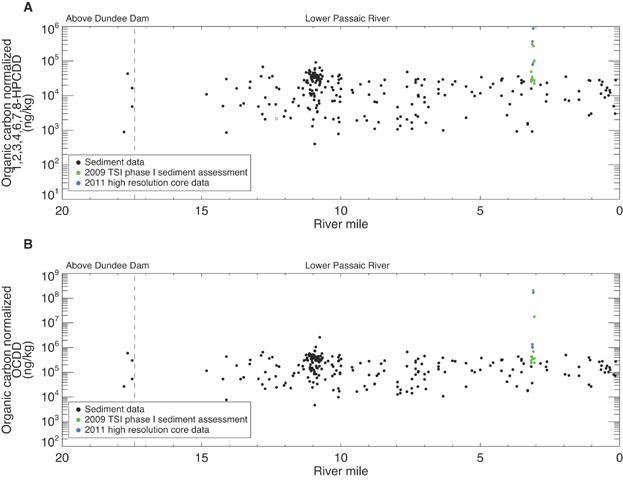
Spatial distribution of organic carbon normalized (A) 1,2,3,4,6,7,8-heptachlorodibenzodioxin (1,2,3,4,6,7,8-HpCDD) and (B) Octachlorodibenzodioxin (OCDD) concentrations in sediment segments with peak 2,3,7,8-tetrachlorodibenzodioxin concentration. Nondetect chemical concentrations were plotted as open symbols at one-half of the method detection limit. Samples with nondetect organic carbon concentrations were excluded from the analysis. TSI = Tierra Solutions.

Finally, 1,2,3,4,6,7,8-HpCDD and OCDD were excluded from fingerprinting analyses based on source considerations and contaminant patterns in the data. Ubiquitous combustion process sources such as wood fires and vehicle exhaust produce a high fraction of the more highly chlorinated dioxin congeners and are common sources of 1,2,3,4,6,7,8-HpCDD and OCDD in the environment 35–39. Also, OCDD may originate from weathering of pentachlorophenol 38,39. These congeners may consequently dominate regional PCDD/F fingerprints. In the lower Passaic River region, 1,2,3,4,6,7,8-HpCDD and OCDD comprise greater than 80% of the 17 2,3,7,8-substituted congener weight in sediment samples collected upstream of the Dundee Dam, and 1,2,3,4,6,7,8-HpCDD and OCDD concentrations in the sediment segments containing the peak 2,3,7,8-TCDD concentrations are relatively uniform throughout the lower Passaic River and similar to those measured upstream of Dundee Dam (with the exception of the phase 1 removal area footprint where elevated concentrations indicate an influence from the site source [Figure 1]). These patterns indicate that substantial regional sources of 1,2,3,4,6,7,8-HpCDD and OCDD exist and, consequently, are not useful tracers of the site even though the phase 1 removal area cores indicate that the site was a source of these congeners to the lower Passaic River. As such, 1,2,3,4,6,7,8-HpCDD and OCDD were excluded from our fingerprinting analyses. This approach is consistent with other fingerprinting investigations. Barabas et al. 35 excluded OCDD from their study of Passaic River dioxin and furan patterns given its ubiquitous nature, which complicated distinguishing among end members by masking differences between other congeners. Bopp et al. 4 suggest an atmospheric OCDD signal based on regional patterns. Towey et al. 40 found high quantities of 1,2,3,4,6,7,8-HpCDD and OCDD to be a common factor in regional background sources in the Tittabawassee River and that relative fractions of lesser chlorinated PCDD/Fs were the most instrumental in distinguishing sources.

The average 15-congener fingerprint for the 1990/1992 site data (discussed in *Comparison of PCDD/F patterns from the Lister Avenue site and the lower Passaic River sediments immediately adjacent to the Lister Avenue site*) shows a PCDD/F pattern that is dominated by 2,3,7,8-TCDD and octachlorodibenzofuran (OCDF). Similar to 1,2,3,4,6,7,8-HpCDD and OCDD, OCDF can also be a common nonpoint source congener [37–39]; however, OCDF was retained in the analysis because of its prevalence at elevated concentrations during the time of peak 2,3,7,8-TCDD discharges from the site, whereas present-day concentrations are more similar to regional levels.

## RESULTS AND DISCUSSION

### 2011 HRC sampling results

**Figure 2:**
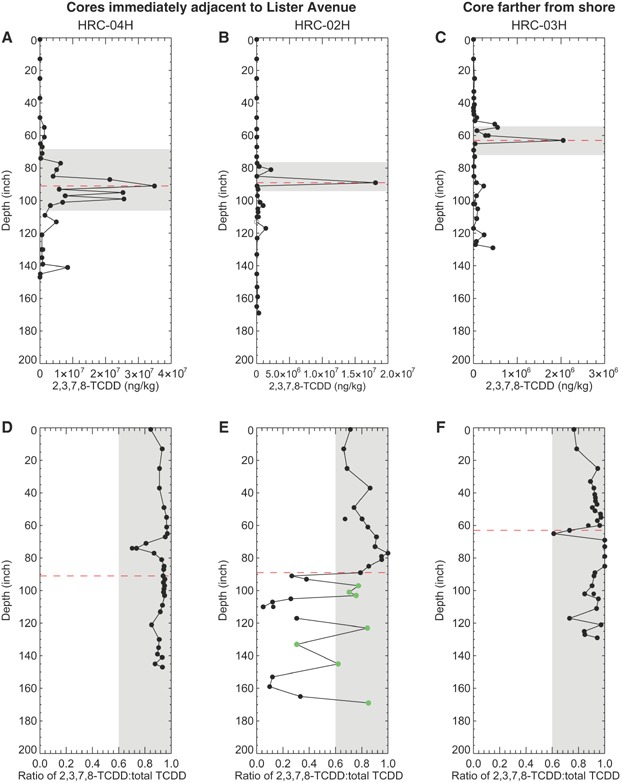
Depth profiles of 2,3,7,8-tetrachlorodibenzodioxin (2,3,7,8-TCDD) concentrations (A–C) and 2,3,7,8-TCDD to total TCDD (D–F) for the 2011 high-resolution core (HRC) collected immediately adjacent to the Lister Avenue Site. (A–C) Horizontal shaded areas represent estimated sediment horizons between 1950 and 1960 based on cesium-137 data. (D–F) Vertical shaded intervals represent a range of ratios between 0.6 and 1.0; horizontal dashed lines represent the sediment depth with peak 2,3,7,8-TCDD concentration; green symbols represent values potentially compromised by poor recovery of TCDD congeners.

In January 2011, 3 sediment HRCs were collected from within the phase 1 removal footprint within the lower Passaic River immediately adjacent to the site in order to evaluate the timing, magnitude, and signature of PCDD/F discharges from the site (Supplemental Data, Figure S1). The vertical distributions of 2,3,7,8-TCDD concentrations measured in the select sediment intervals from these cores are shown in Figure 2A–C. Superimposed on these concentration profiles are horizontal shaded bands that represent the estimated 1950 to 1960 period, the time frame coinciding with peak discharges from the site. This period was identified using Cs-137 measurements as time markers; a consequence of nuclear weapons testing, Cs-137 deposition in the environment started in approximately 1952, had accumulated to measurable amounts in soils beginning in approximately 1954 41,42, and peaked in concentration in approximately 1963, the year of maximum atmospheric fallout 43. Using the 1954 and 1963 markers, the average deposition rate for the 1954 to 1963 period was determined and applied to the core intervals to estimate the 1950 to 1960 period.

All 3 cores exhibit a typical depositional profile, with peak 2,3,7,8-TCDD concentrations at depth and declining concentrations with distance above the peak (i.e., moving up the sediment column toward the core surface). Peak 2,3,7,8-TCDD concentrations range from 2 050 000 to 34 900 000 ng/kg (or 2.05–34.9 mg/kg), which represent the highest concentrations measured in the lower Passaic River sediments. The core situated farthest from shore (HRC-03H; see Figure 2C) has a lower peak concentration than the nearshore cores, presumably reflecting the trapping and dilution processes that occurred as discharges were transported away from the site. In all 3 cores, peak 2,3,7,8-TCDD concentrations date to the 1950s, as evidenced by the horizontal shaded bands established through radioisotope age dating (Figure 2A–C). This peak date is consistent with the known 2,3,7,8-TCDD discharge history from the site 9,44. Also evident in these cores are secondary 2,3,7,8-TCDD peaks, roughly at 80 inches (HRC-04H and HRC-02H; see Figure 2A, B) and 55 inches (HRC-03H; see Figure 2C) below the sediment surface. The timing of these secondary peaks generally coincides with the February 1960 trichlorophenol autoclave explosion at the site that released 2,3,7,8-TCDD and other compounds to the surrounding area 44,45. The decline in 2,3,7,8-TCDD concentrations after this explosion presumably reflects smaller mass discharge from the site after the replacement of the main processing facility in 1961 and the end of 2,4,5-trichlorophenoxyacetic acid production in 1969 35,44, as well as a natural recovery of surface sediments. Surface 2,3,7,8-TCDD concentrations in the 3 cores range from 418 ng/kg to 776 ng/kg, roughly 3.0 to 4.5 orders of magnitude lower than the peak 2,3,7,8-TCDD concentrations.

**Figure 3:**
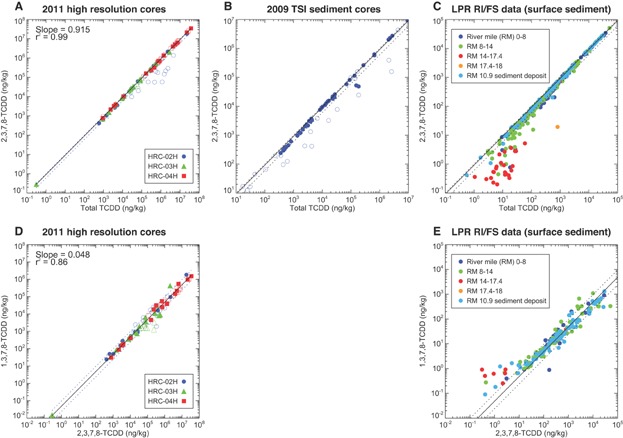
Cross-plots of 2,3,7,8-tetrachlorodibenzodioxin (2,3,7,8-TCDD) to total TCDD concentrations (A–C) for the 2011 high-resolution cores (HRC; A) and 2009 Tierra Solutions (TSI) cores (B) collected immediately adjacent to the Lister Avenue Site and for the surface sediments of the lower Passaic River (C), and cross-plots of 1,3,7,8-TCDD to 2,3,7,8-TCDD concentrations (D, E) for the 2011 HRCs collected immediately adjacent to the Lister Avenue Site (D) and surface sediments of the lower Passaic River (E). (A–C) Solid lines represent the average ratio of 0.92 based on the 2011 HRC data; dashed lines represent the range in the ratios of 0.6 to 1.0; filled symbols denote samples at or above peak 2,3,7,8-TCDD; open symbols denote samples below peak 2,3,7,8-TCDD depth interval. (D, E) Solid lines represent the average ratio of 0.048 from the 2011 HRC data; dashed lines represent a factor of 2 around the average of 0.048 from the 2011 HRC data; filled symbols denote samples at or above peak 2,3,7,8-TCDD depth interval; open symbols denote samples below peak 2,3,7,8-TCDD. LPR = lower Passaic River; RI/FS = remedial investigation and feasibility study; RM = river mile; TSI = Tierra Solutions.

Following studies that used the ratio of 2,3,7,8-TCDD to total TCDD as a tracer for the site 5,11,24, we examined this ratio in the 2011 HRC data. A cross-plot of 2,3,7,8-TCDD to total TCDD concentration from the 2011 HRCs data is shown in Figure 3A. With an average ratio (or slope, shown as a solid line) of 0.92 and the vast majority of the samples falling within a range of 0.6 to 1.0 (shown as dashed lines), 2,3,7,8-TCDD concentrations show a tight correlation to total TCDD. The high ratios are consistent with those measured in the 1990/1992 site samples (4 of the 5 samples had ratios between 0.91 and 0.98; 1 sump residue sample [A5] had a ratio of 0.41), soil samples in the vicinity of the site (0.86–0.98) 13,14, and sediments in much of the lower Passaic River (see *Comparison of PCDD/F patterns from the Lister Avenue site and sediments throughout the lower Passaic River* and Israelsson et al. 23) and Newark Bay (0.71 and 0.86) 5. A number of samples from core HRC-02H have ratios less than 0.6 (see blue open circles in Figure 3A). Examination of the vertical profiles of the 2,3,7,8-TCDD to total TCDD ratios in these cores (Figure 2D–F) indicates that the 2,3,7,8-TCDD to total TCDD ratios that are less than approximately 0.6 are all associated with samples collected below the peak 2,3,7,8-TCDD concentration in core HRC-02H (see Figure 2E). The intervals at and above the peak 2,3,7,8-TCDD concentration in core HRC-02H are all above 0.6, whereas those in cores HRC-04H and HRC-03H (Figure 2D, F, respectively) are consistently near or above 0.9 throughout the sediment column.

The ratios of 2,3,7,8-TCDD to total TCDD from the 2009 Tierra Solutions cores exhibit a similar pattern (Figure 3B); most of the ratios are above 0.6, and the instances where ratios of less than 0.6 are observed are associated with samples collected below the peak 2,3,7,8-TCDD concentration. The cause for the lower ratios below the peak 2,3,7,8-TCDD concentration in a subset of the 2009 Tierra Solutions cores and 2011 HRCs is not known, but they could reflect changes in the manufacturing processes at the site over time and/or signatures of different discharges from the site. This is supported by the spatial patterns observed within the phase 1 removal footprint; cores with 2,3,7,8-TCDD to total TCDD ratios less than 0.6 below the peak 2,3,7,8-TCDD concentration are almost exclusively found in the downstream half of the footprint that is adjacent to the former chemical manufacturing building, whereas cores with 2,3,7,8-TCDD to total TCDD ratios greater than 0.6 below the peak 2,3,7,8-TCDD concentration are found in the upstream half of the footprint that is adjacent to the former processing building (see Supplemental Data, Figure S1). An overall assessment is provided by looking at the total mass in the cores. The estimated ratio of 2,3,7,8-TCDD mass to total TCDD mass within the phase 1 removal footprint exceeds 0.8, which is well above the ratio of less than 0.06 that Chaky 5 attributed to background sources. Thus, the 2009 and 2011 core data sets support the use of 2,3,7,8-TCDD to total TCDD as a tracer of the site discharge in lower Passaic River sediments 5,11,24.

In addition to 2,3,7,8-TCDD concentrations and the 2,3,7,8-TCDD to total TCDD ratios, correlations between 2,3,7,8-TCDD and other PCDD/F congener concentrations with similar environmental properties were examined to investigate the potential for other PCDD/F congener ratios to serve as a tracer of the site source. The ratio of 1,3,7,8-TCDD to 2,3,7,8-TCDD was identified as a possible tracer. A cross-plot of 1,3,7,8-TCDD to 2,3,7,8-TCDD concentration from the 2011 HRCs data is shown in Figure 3D. Concentrations of 1,3,7,8-TCDD and 2,3,7,8-TCDD are strongly correlated, with an average ratio (or slope, shown as solid line in Figure 3D) of 0.048 and a correlation coefficient of 0.86. In most instances, these values are within a factor of 2 of the average ratio (factors of 2 shown as dashed lines in Figure 3D). Given this limited variability despite the 5 orders of magnitude variability in measured 2,3,7,8-TCDD concentrations in these 2011 HRCs (see Figure 2A–C), the ratio of 1,3,7,8-TCDD to 2,3,7,8-TCDD can be used as another tracer of the site source. The low variability in this ratio throughout the sediment column also suggests a lack of other significant 1,3,7,8-TCDD sources in the region, which, if present, would alter this ratio, as sediments with the site signal would be diluted through mixing with sediments from these 1,3,7,8-TCDD sources.

### Comparison of PCDD/F patterns from the Lister Avenue site and the lower Passaic River sediments immediately adjacent to the Lister Avenue site

Building on prior studies that identified the ratio of 2,3,7,8-TCDD to total TCDD as a tracer for the site source 5,11,24, we look herein at the relationship between PCDD/F congener and homolog patterns measured in samples collected from the site and lower Passaic River sediments. For the congener fingerprinting, the comparisons consider 15 of the 17 2,3,7,8-substituted congeners; 1,2,3,4,6,7,8-HpCDD and OCDD are excluded because of their ubiquitous presence throughout the region (see *Materials and Methods* discussion). For the same reason, total HpCDD and OCDD are excluded from the homolog fingerprinting comparisons.

**Figure 4:**
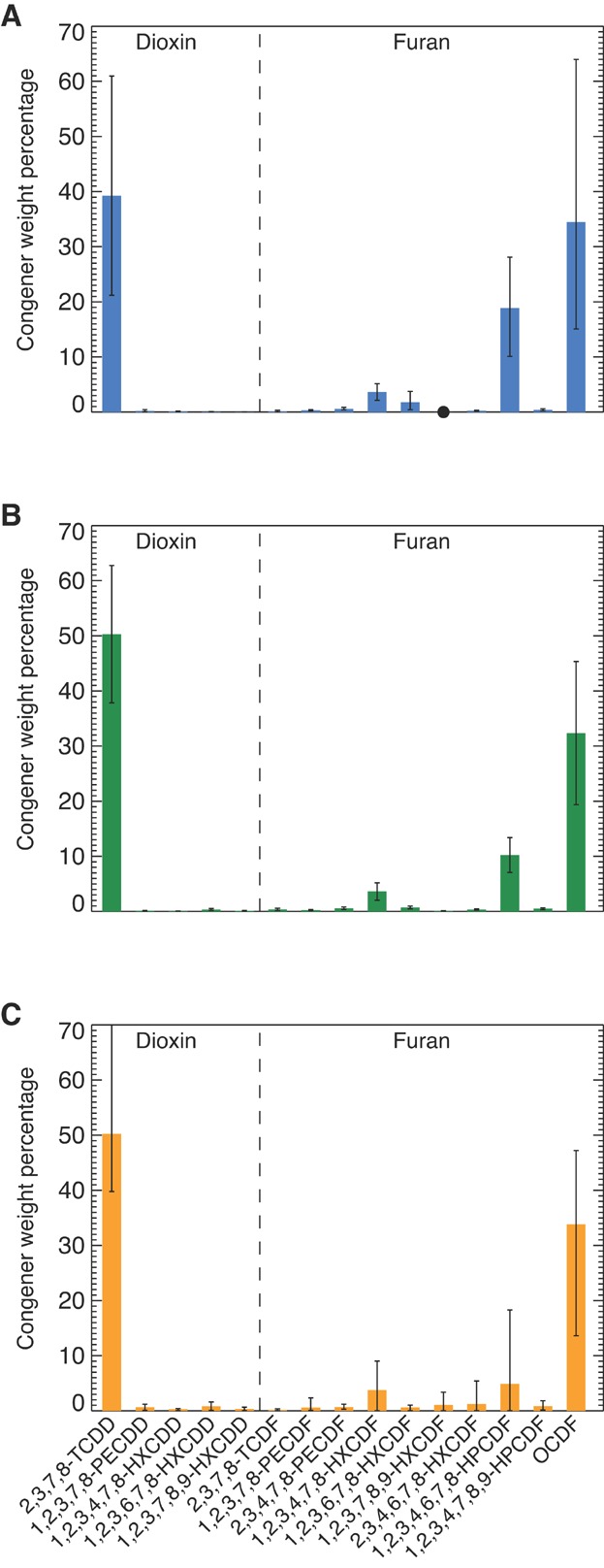
Average polychlorinated dibenzodioxin/polychlorinated dibenzofuran congener composition in (A) 2011 high-resolution cores at the local peak 2,3,7,8-tetrachlorodibenzodioxin (2,3,7,8-TCDD) concentration, (B) 2009 Tierra Solutions cores at the local peak 2,3,7,8-TCDD concentration, and (C) 1990/1992 Lister Avenue Site samples. Error bars represent ranges for (A) and (C) and 2 times the standard error of the mean for (B). HPCDF = heptachlorodibenzofuran; HXCDD hexachlorodibenzodioxin; HXCDF = hexachlorodibenzofuran; OCDF = octachlorodibenzofuran; PECDD = pentachlorodibenzodioxin; PECDF = pentachlorodibenzofuran; 2,3,7,8-TCDD = 2,3,7,8-tetrachlorodibenzodioxin; TCDF = tetrachlorodibenzofuran; TSI = Tierra Solutions.

The average PCDD/F congener compositions in the 5 1990/1992 site samples (i.e., A1–A5), the 11 2009 Tierra Solutions cores, and the 3 2011 HRCs are compared in Figure 4. In each instance, the signature represents arithmetic averages of the weight percentage for each of the 15 2,3,7,8-substituted PCDD/F congeners. The 2009 Tierra Solutions and 2011 HRC values are from the sediment intervals with the peak 2,3,7,8-TCDD concentrations. The error bars in the 2009 Tierra Solutions core fingerprints represent 2 standard errors around the mean, whereas those for the 2011 HRCs and site samples denote the range of values. Ranges are presented for the 2011 HRC and site fingerprint because of the small sample size (i.e., 5 or fewer data points).

The 2011 HRC fingerprint is dominated by the 2,3,7,8-TCDD component, which, on average, represents approximately 39% of the total of the 15 PCDD/F congeners (Figure 4A). Following 2,3,7,8-TCDD, the most prevalent congeners in decreasing order are OCDF (representing ∼34% of the total), 1,2,3,4,6,7,8-heptachlorodibenzofuran (1,2,3,4,6,7,8-HpCDF, representing ∼19% of the total), and 1,2,3,4,7,8-hexachlorodibenzofuran (1,2,3,4,7,8-HxCDF, representing ∼4% of the total). The 2009 Tierra Solutions fingerprint exhibits a similar pattern, although it is slightly enriched in 2,3,7,8-TCDD and lower in 1,2,3,4,6,7,8-HxCDF relative to the 2011 HRC signature (Figure 4B).

The site fingerprint (Figure 4C) comes from site soils and sump residue samples, which are the result of residual contamination during the manufacturing process. As such, the fact that concentrations in these samples are lower than those measured in the 2009 Tierra Solutions cores and 2011 HRCs cores is expected—these samples do not necessarily reflect the concentration of what was discharged to the river. They do, however, provide an indication of the composition of what was discharged to the river. Although the composition varies among these samples (fingerprints in the 5 site samples used in the present evaluation are provided in Supplemental Data, Figure S2), the average PCDD/F congener pattern of these samples closely matches those from the 2009 Tierra Solutions cores (Figure 4B) and 2011 HRCs (Figure 4A). The dominant congeners are 2,3,7,8-TCDD (∼50%) and OCDF (∼34%), followed by 1,2,3,4,6,7,8-HpCDF and 1,2,3,4,7,8-HxDCF at approximately 5% and 4%, respectively. This similarity confirms the expected link between the PCDD/Fs from the site and those found in the lower Passaic River sediments immediately adjacent to the site (i.e., within the phase 1 removal area), as well as the utility of the signature as a fingerprint in sediments impacted by the site source.

### Comparison of PCDD/F patterns from the Lister Avenue site and sediments throughout the lower Passaic River

**Figure 5:**
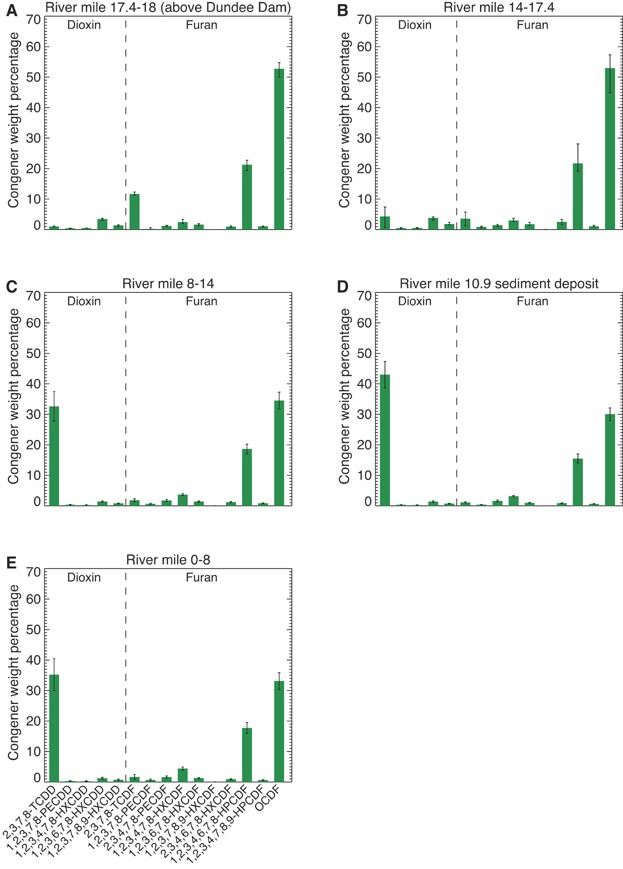
Average polychlorinated dibenzodioxin/polychlorinated dibenzofuran congener composition at local peak 2,3,7,8-TCDD concentration in lower Passaic River sediments from (A) RM 17.4 to RM 18, (B) RM 14 to RM 17.4, (C) RM 8 to RM 14 (excluding RM 10.9 data), (D) RM 10.9 sediment deposit, and (E) RM 0 to RM 8. (A,B) Error bars represent ranges of the data, resulting from a limited number of data points in these bins. (C–E) Error bars represent 2 times the standard error of the mean. RM = river mile; HPCDF = heptachlorodibenzofuran; HXCDD hexachlorodibenzodioxin; HXCDF = hexachlorodibenzofuran; OCDF = octachlorodibenzofuran; PECDD = pentachlorodibenzodioxin; PECDF = pentachlorodibenzofuran; 2,3,7,8-TCDD = 2,3,7,8-tetrachlorodibenzodioxin; TCDF = tetrachlorodibenzofuran.

Comparisons between PCDD/F congener patterns in the 2011 HRCs and sediments from throughout the lower Passaic River are presented in Figure 5 using averages of the congener patterns observed in the peak 2,3,7,8-TCDD concentration intervals of samples within each designated reach of the river. The PCDD/F congener compositions in RM 0 to RM 8 (Figure 5E) and RM 8 to RM 14 (Figure 5C) sediments are remarkably similar and closely resemble those of the 2011 HRCs (Figure 4A) and 2009 Tierra Solutions cores (Figure 4B). They are dominated by 2,3,7,8-TCDD and OCDF, each accounting for approximately 33% to 35% of the total, followed by approximately 18% to 19% of 1,2,3,4,6,7,8-HpCDF, 4% of 1,2,3,4,7,8-HxCDF, and trace amounts of the remaining PCDD/F congeners. These 4 PCDD/F congeners are also observed in the RM 10.9 sediment signature (Figure 5D) in roughly the same proportions as in the 2011 HRCs (Figure 4A), although a slight enrichment of 2,3,7,8-TCDD is noted in this deposit relative to those found in the RM 8 to RM 14 sediments (Figure 5C). Differences in the proportion of 2,3,7,8-TCDD in the RM 10.9 and RM 8 to RM 14 samples may reflect inherent variability and/or possible localized small contributions of other PCDD/F sources. The notion of other less significant PCDD/F sources to the lower Passaic River has been noted by other investigators such as Huntley et al. 19, Hansen 10, and Garvey et al. 20, who suggested the possibility of a second 2,3,7,8-TCDD source in the vicinity of the Third River (near RM 11). However, soil samples from the Givaudan facility, the site identified by Hansen 10 as the second 2,3,7,8-TCDD source, exhibit a different 15-congener fingerprint from the Lister Avenue site source. The Givaudan facility fingerprint is comprised primarily of 2,3,7,8-TCDD, along with pentachlorodibenzodioxin (PeCDD), hexachlorodibenzodioxin (HxCDD), and low proportions of PCDF congeners (Supplemental Data, Figure S3). In contrast, the Lister Avenue site fingerprint contains 2,3,7,8-TCDD, lacks significant proportions of PeCDD and HxCDD congeners, and contains a significant fraction of PCDF congeners such as 1,2,3,4,6,7,8-HpCDF and OCDF, which are found in comparable proportions in the RM 10.9 and lower Passaic River sediments. Although the Givaudan facility signature contains 2,3,7,8-TCDD, contributions from this source, if any, must be small relative to the site source given that higher proportions of the PeCDD and HxCDD congeners seen in the Givaudan facility fingerprint are not observed in the RM 10.9 and other lower Passaic River sediments. To the extent that other sources with a fingerprint similar to that of the site source exist in this region, their contributions to 2,3,7,8-TCDD levels in the lower Passaic River sediments relative to the site source would not be discernable.

The PCDD/F congener fingerprint in sediments from RM 14 to RM 17.4 (Figure 5B) resembles that found in sediments above the Dundee Dam (i.e., RM 17.4–18; see Figure 5A). In both RM 14 to RM 17.4 and RM 17.4 to RM 18, the pattern features a dominant OCDF component (∼53%) and relatively lower fractions of 2,3,7,8-TCDD (∼4% in RM 14–17.4 and 1% in RM 17.4–18). A greater proportion of other PCDD/F congeners, such as 2,3,7,8-tetratchlorodibenzofuran and 1,2,3,6,7,8-hexachlorodibenzodioxin (1,2,3,6,7,8-HxCDD), is also noted in the more upstream samples (i.e., RM 14–17.4 and RM 17.4–18). The differences between these PCDD/F congener fingerprints and the site fingerprint indicate that the site influence on sediments is largely limited to the lower 14 miles of the lower Passaic River (excluding, for the purposes of the present discussion, Newark Bay). The slightly higher proportion of 2,3,7,8-TCDD observed in the RM 14 to RM 17.4 bin (relative to upstream) suggests some site influence on this region. This is consistent with the physical understanding of the system, which indicates that transport associated with tidal resuspension and salinity intrusion would have moved contaminants from the site upstream 23,46. As discussed in Chant et al. 46 and Israelsson et al. 23, during low-flow conditions, a net upstream transport of solids is observed within the estuarine portion of the lower Passaic River, attributable to the estuarine circulation and tidal pumping. Although the salt front typically resides in the lower 8 miles of the lower Passaic River, it can extend above RM 14 under extreme, persistent low-flow conditions (such as those experienced during the drought conditions in the mid-1960s). The upstream solids transport implies an upstream contaminant transport that is consistent with the lower Passaic River sediment data patterns, and moreover, net upstream contaminant transport is plausible above the salt front in the presence of longitudinal gradients in contaminant concentration (see Israelsson et al. 23 for additional discussion).

**Figure 6:**
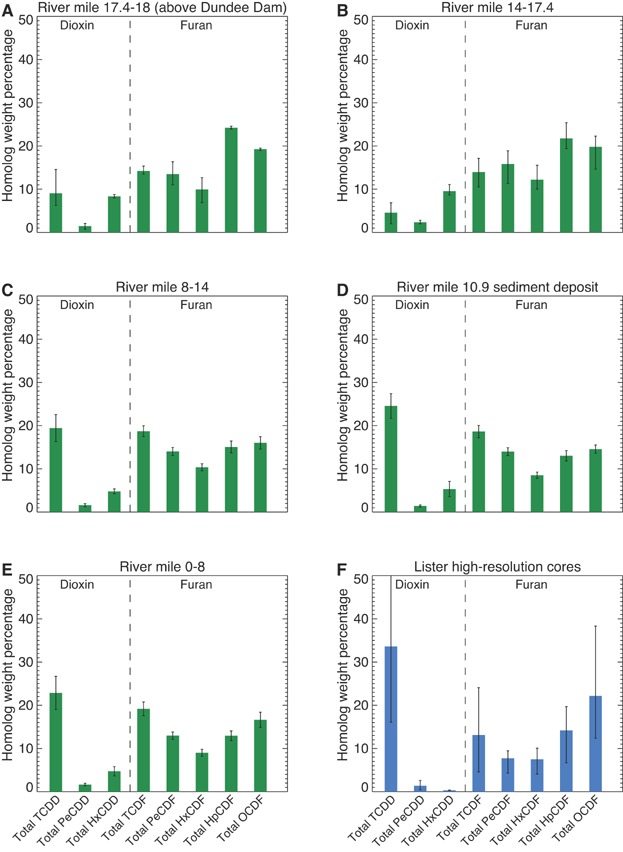
Average polychlorinated dibenzodioxin/polychlorinated dibenzofuran homolog composition at local peak 2,3,7,8-TCDD concentration in sediments from (A) RM 17.4 to RM 18, (B) RM 14 to RM 17.4, (C) RM 8 to RM 14 (excluding RM 10.9 data), (D) RM 10.9 sediment deposit, (E) RM 0 to RM 8, and (F) 2011 high-resolution cores immediately adjacent to the Lister Avenue Site. (A,B,F) Error bars represent ranges of the data, resulting from a limited number of data points in these bins. (C–E) Error bars represent 2 times the standard error to the mean. RM = river mile; HpCDF = heptachlorodibenzofuran; HxCDD hexachlorodibenzodioxin; HxCDF = hexachlorodibenzofuran; OCDF = octachlorodibenzofuran; PeCDD = pentachlorodibenzodioxin; PeCDF = pentachlorodibenzofuran; 2,3,7,8-TCDD = 2,3,7,8-tetrachlorodibenzodioxin; TCDF = tetrachlorodibenzofuran.

The PCDD/F homolog composition provides an additional and distinct line of evidence because it is mostly determined by congeners other than the 2,3,7,8-substituted congeners used for the congener fingerprint analysis 37. The homolog fingerprints for the lower Passaic River sediments and the 2011 HRCs are compared in Figure 6; the 2009 Tierra Solutions cores are not shown because the full suite of PCDD/F homolog information was not reported for these samples. The RM 0 to RM 8 (Figure 6E), RM 8 to RM 14 (Figure 6C), and RM 10.9 (Figure 6D) sediment deposit and sediments immediately adjacent to the site (Figure 6F) share similar characteristics: a relatively large proportion of total TCDD and a *U*-shaped distribution for the PCDF homologs, with the highest weight percentages for OCDF and tetrachlorodibenzofuran and the lowest for total hexachlorodibenzofuran (HxCDF). Only small amounts of total PeCDD and total HxCDD are observed in these samples. Consistent with the PCDD/F congener patterns, a slight enrichment in total TCDD is observed in the RM 10.9 sediments relative to those in the RM 0 to RM 8 and RM 8 to RM 14 sediments. In contrast, the homolog composition in the RM 17.4 to RM 18 and RM 14 to RM 17.4 sediments (Figure 6A and B, respectively) differs substantially from the 2011 HRC, RM 0 to RM 8, and RM 8 to RM 14 sediment fingerprints (Figure 6F, E, and C, respectively). In the RM 17.4 to RM 18 and RM 14 to RM 17.4 sediments, the total homolog pattern shows strong total HpCDF and OCDF influences, followed by the other PCDF homologs (total tetrachlorodibenzofuran, total pentachlorodibenzofuran, and total HxCDF). The PCDDs represent only a small fraction of the overall composition (∼16–19%), with total TCDD ranging from approximately 5% to 9%. This total TCDD fraction is much lower than that observed in the RM 0 to RM 8, RM 8 to RM 14, RM 10.9, and 2011 HRC sediments (19–34%). These comparisons are consistent with the PCDD/F congener patterns and support our conclusions regarding the historical importance of the site source on 2,3,7,8-TCDD levels in lower Passaic River sediments.

**Figure 7:**
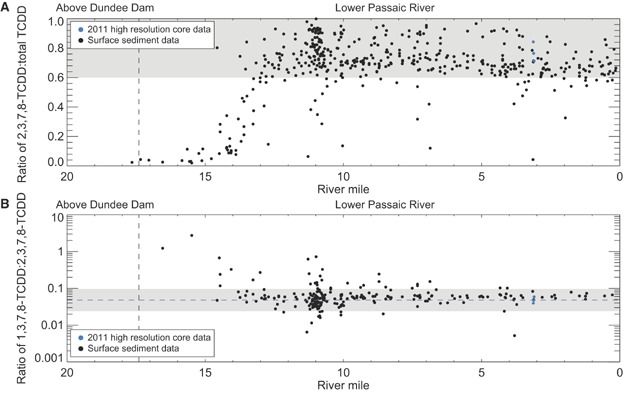
Spatial distribution of (A) 2,3,7,8-tetrachlorodibenzodioxin (2,3,7,8-TCDD) to total TCDD ratios and (B) 1,3,7,8-TCDD to 2,3,7,8-TCDD ratios in the surface sediment of lower Passaic River. (A) The shaded interval represents a range of ratios between 0.6 and 1. (B) The dashed line and shaded interval represent the average ratio of 0.048 from the 2011 high-resolution cores and a factor of 2 around the average, respectively.

In addition to the PCDD/F congener and homolog compositions, the distributions of 2 congener ratios were examined: 2,3,7,8-TCDD to total TCDD, as has been used by other investigators 5, and 1,3,7,8-TCDD to 2,3,7,8-TCDD (as discussed in *2011 HRC sampling results*). In the present study, we focus on the lower Passaic River surface sediments to take advantage of several remedial investigation and feasibility study data sets that contain information for all 136 PCDD/F congeners (i.e., 2009 remedial investigation field sampling plan 2 benthic sediment sampling program, 2011/2012 RM 10.9 sediment data, 2012 supplemental sediment sampling program, and 2013 second supplemental sediment sampling program); all other remedial investigation and feasibility study data sets measured only the 17 2,3,7,8-substituted congeners. The 2 ratios are plotted spatially in Figure 7 and as cross-plots in Figure 3C and E.

The majority of the 2,3,7,8-TCDD to total TCDD ratios in the surface sediment vary between 0.6 and 1.0 throughout the lower 13 miles of the lower Passaic River (Figures 7A and 3C), which reinforces the conclusion from the PCDD/F congener and homolog patterns that the 2,3,7,8-TCDD contamination in this region originated principally from the site. The fact that these elevated 2,3,7,8-TCDD to total TCDD ratios persist in present-day lower Passaic River surface sediments decades after site discharges ceased is attributed to older sediments with extremely high 2,3,7,8-TCDD concentrations being mixed with and buried by low-concentration solids, which has diluted sediment 2,3,7,8-TCDD concentrations over time but has maintained the 2,3,7,8-TCDD to total TCDD ratios associated with the original site discharges (i.e., this source still dominates the 2,3,7,8-TCDD concentrations). At approximately RM 13, the congener ratio drops below 0.6, the result of mixing between the site source signal and upstream sources. The congener ratio drops below 0.2 by RM 15 and reaches 0.04 to 0.05 by RM 16, a ratio consistent with what is typically seen in wastewater and atmospheric sources 5. Below RM 14, surface ratios below 0.6 are primarily associated with low 2,3,7,8-TCDD concentrations (<100 ng/kg; Figure 3C), presumably reflecting the influence of regional sources of TCDD congeners other than 2,3,7,8-TCDD that becomes evident in low concentrations. This spatial pattern in the 2,3,7,8-TCDD to total TCDD ratio indicates a site influence that declines in strength moving upstream of RM 13 and appears nominal above RM 15. This finding is consistent with the approximate breakpoint of RM 14 for the site influence that was identified in Israelsson et al. 23 using a smaller data set on the basis of ratios at the 2,3,7,8-TCDD peak depth as well as the magnitude of the 2,3,7,8-TCDD peak concentration and estimated mass inventory. The existence of elevated concentrations and 2,3,7,8-TCDD to total TCDD ratios between RM 14 and RM 15 in the 2013 second supplemental sediment sampling program data set included herein indicates that the breakpoint is somewhat above RM 14 but remains consistent with the physical upstream transport mechanisms within the lower Passaic River 23. Nevertheless, the declining surface sediment 2,3,7,8-TCDD to total TCDD ratios above RM 14 are consistent with the findings of the preceding analysis of PCDD/F congener and homolog patterns (see above).

Similarly, the 1,3,7,8-TCDD to 2,3,7,8-TCDD ratios in surface sediments are relatively uniform throughout the lower 14 miles of the lower Passaic River, existing within a tight range that is consistent with that observed in the 2011 HRCs (shown as the horizontal shading in Figure 7B and dashed lines in Figure 3E). The greater variability in this ratio observed in the RM 10.9 region is likely a statistical artifact of the greater density of samples collected within this smaller area. Upstream of approximately RM 14 the 1,3,7,8-TCDD to 2,3,7,8-TCDD ratio increases. The similarity of the ratio between the 2011 HRCs and lower Passaic River surface sediments and the relatively uniform spatial pattern observed throughout the lower Passaic River provides additional support for the conclusion that the site is the dominant source of 2,3,7,8-TCDD to approximately the lower 14 miles of the lower Passaic River.

## SUMMARY AND CONCLUSION

The sediments of the lower 14 miles of the lower Passaic River contain a relatively uniform PCDD/F composition that resembles the composition of sediments adjacent to the Lister Avenue site, which in turn matches the composition of samples taken on the upland site. The match has been demonstrated using several fingerprints: 15-congener PCDD/F composition, PCDD/F homolog composition, 1,3,7,8-TCDD to 2,3,7,8-TCDD ratios, and 2,3,7,8-TCDD to total TCDD ratios. The match strongly indicates that the Lister Avenue site is the dominant source of the examined PCDD/Fs over this region. The upstream influence of the site, which extends to approximately RM 14 (congener ratios indicate a gradually declining influence over the RM 13–15 interval in particular), is consistent with upstream contaminant transport and salinity intrusion considerations, particularly during the peak site discharge period 19,23.

Although the fingerprints given in the present study are useful for linking 2,3,7,8-TCDD contamination in lower Passaic River sediments to the site, the fingerprints themselves may not be directly transferable to source investigation studies in other contaminated systems. This is because the manufacturing and production processes employed at the site resulted in a unique chemical fingerprint; several factors, such as a different set of ingredients and/or different manufacturing temperatures/processes, can result in a different chemical signature. The value of the present analyses, however, is 2-fold. First, the present study demonstrates that the site source fingerprint has been largely conserved over space and time and that fate and transport processes that occur in the system do not substantively alter the source fingerprint. Second, the use of multiple and distinct signatures to link lower Passaic River contamination to the site source provides greater strength to the conclusions drawn from such an analysis. Individually, the fingerprints in the present study suggest such a linkage, but when viewed together, the more robust evaluation provides greater confidence that the linkage exists.

## SUPPLEMENTAL DATA

**Figures S1–S3.** (2.4 MB PDF).

## Data Availability

All data are available on request from the authors (jquadrini@anchorqea.com).

## Supporting Information

All Supplemental Data may be found in the online version of this article.
