# Supplementary material for: Fingerprinting 2,3,7,8-tetrachlorodibenzodioxin contamination within the lower Passaic River
Source: Environ Toxicol Chem. 2015 May 5;34(7):1485–98. doi: 10.1002/etc.2961 (PMC4676307; doi:10.1002/etc.2961)
Supplement: Supplementary file 1 [file etc0034-1485-sd1.pdf]

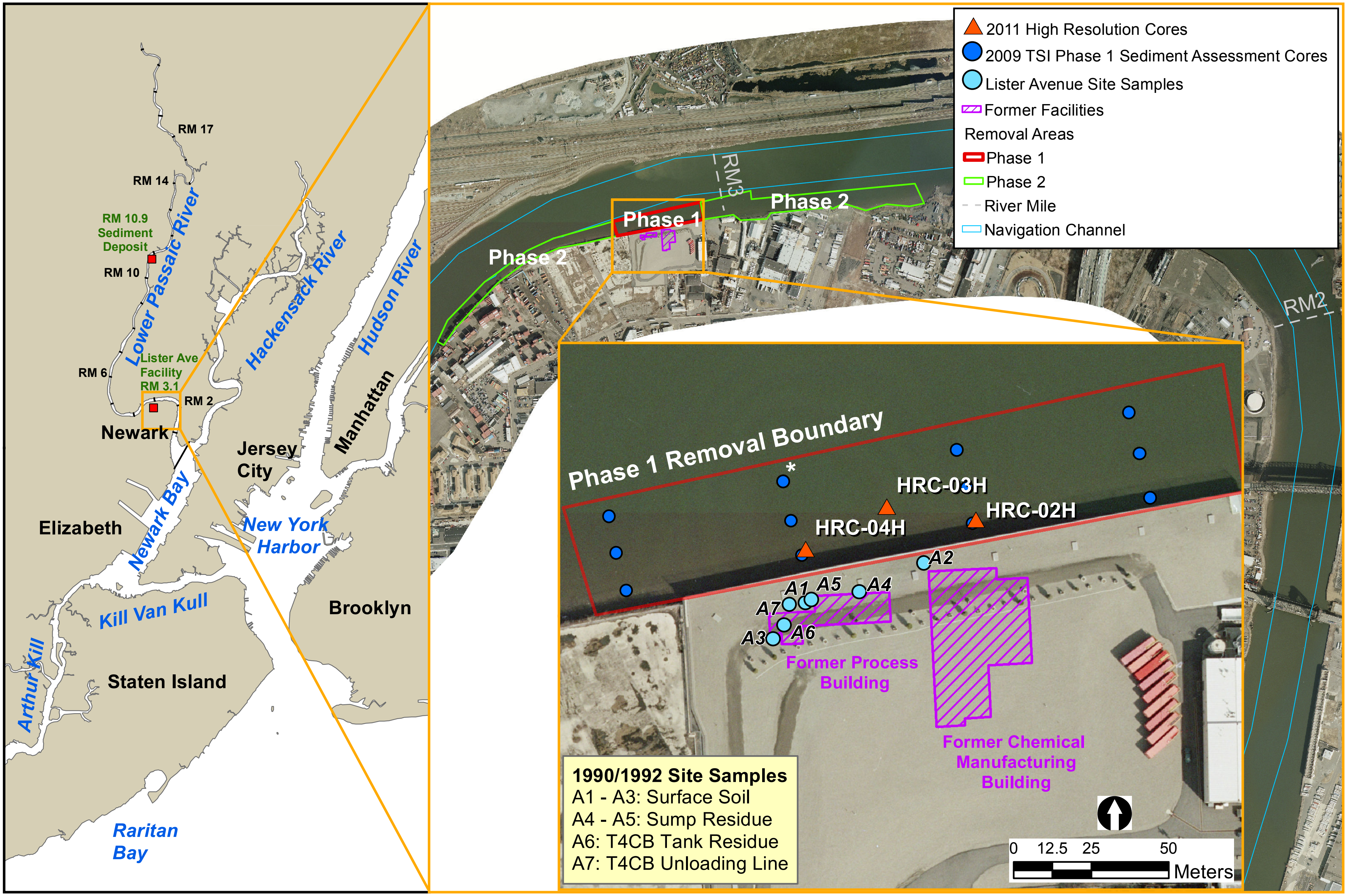

Note:
* Excluded from the 2,3,7,8-TCDD to Total TCDD analysis because the ratios are greater than 1 in the entire core.

Figure S1

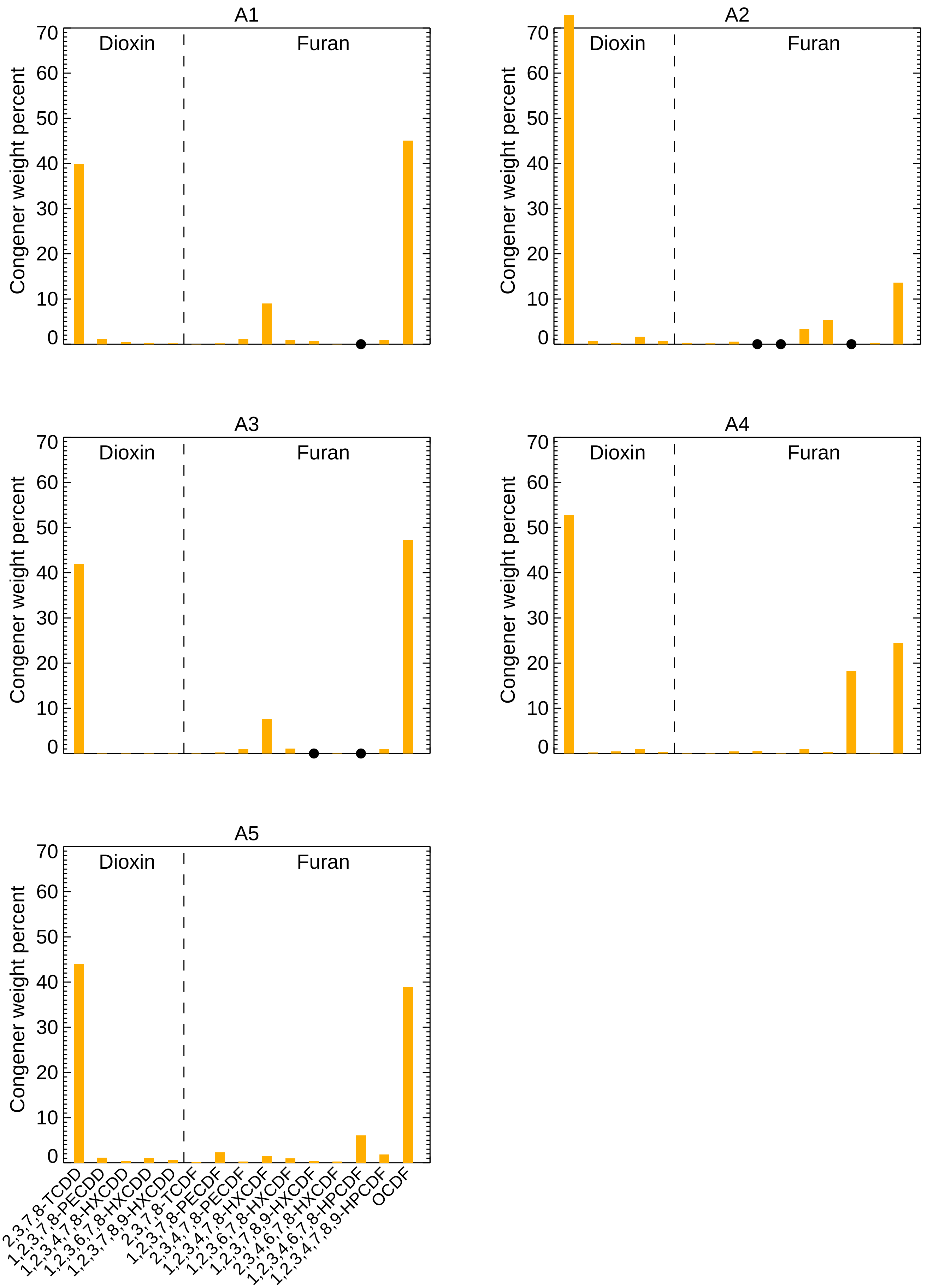
A1
A2
A3
A4
A5
Dioxin
Furan
Congener weight percent
0
10
20
30
40
50
60
70
2,3,7,8-TCDD
1,2,3,7,8-PECDD
1,2,3,4,7,8-HXCDD
1,2,3,6,7,8-HXCDD
1,2,3,7,8,9-HXCDD
2,3,7,8-TCDF
1,2,3,7,8-PECDF
2,3,4,7,8-PECDF
1,2,3,4,7,8-HXCDF
1,2,3,6,7,8-HXCDF
1,2,3,7,8,9-HXCDF
2,3,4,6,7,8-HXCDF
1,2,3,4,6,7,8-HPCDF
1,2,3,4,7,8,9-HPCDF
OCDF

Figure S2

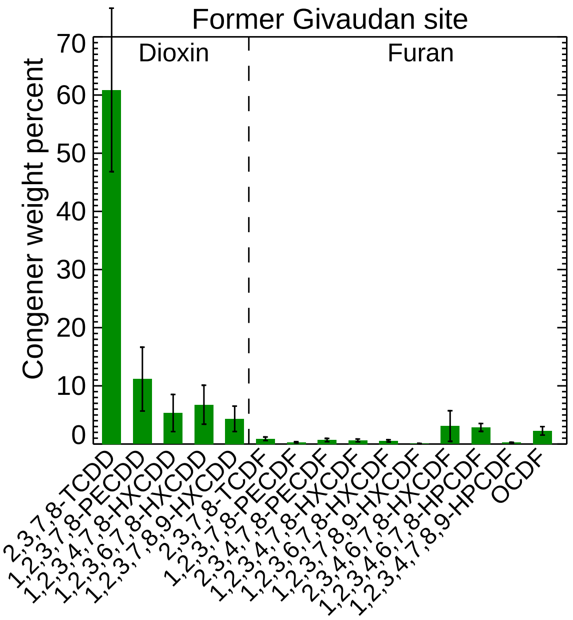
Former Givaudan site
Dioxin
Furan
Congener weight percent
0
10
20
30
40
50
60
70
2,3,7,8-TCDD
1,2,3,7,8-PECDD
1,2,3,4,7,8-HXCDD
1,2,3,6,7,8-HXCDD
1,2,3,7,8,9-HXCDD
2,3,7,8-TCDF
1,2,3,7,8-PECDF
2,3,4,7,8-PECDF
1,2,3,4,7,8-HXCDF
1,2,3,6,7,8-HXCDF
1,2,3,7,8,9-HXCDF
2,3,4,6,7,8-HXCDF
1,2,3,4,6,7,8-HPCDF
1,2,3,4,7,8,9-HPCDF
OCDF

Figure S3
